# Meningiomas in Ancient Human Populations

**DOI:** 10.3390/cancers14041058

**Published:** 2022-02-19

**Authors:** Della Collins Cook, Marie Elaine Danforth

**Affiliations:** 1 Department of Anthropology, Indiana University, Bloomington, IN 47405, USA; 2 School of Social Sciences and Global Studies, University of Southern Mississippi, Hattiesburg, MS 39406, USA; m.danforth@usm.edu

**Keywords:** meningioma, hyperostosis, differential diagnosis, paleopathology, cranial tumors

## Abstract

**Simple Summary:**

Meningiomas are the most common tumor of the central nervous system but are rare in the paleopathological record. Although they are technically a soft tissue phenomenon, they do leave various lesions on the skeletons, including thickened bone adjacent to the tumor and vascular impression changes. A review of the literature of health in past populations revealed some 43 cases of lesions identified by the original authors as meningioma. These cases are considered in terms of the appearance of the lesions as well as alternative diagnoses. The age distribution fits modern demographic patterns for meningioma patients but the sex distribution is roughly opposite of current patterns. It is suggested that meningiomas should be considered more often in differential diagnoses in ancient people.

**Abstract:**

Meningiomas are the most common tumor of the central nervous system and can result in skeletal manifestations, including hyperostosis of the adjacent cranial bone, enostoses, depressions, and enhanced vascular impressions. However, their identification in the paleopathological literature has been rare and few cases have received broad acceptance of the diagnosis. A review of the literature identified some 43 cases in which individuals were argued to have suffered from meningiomas. Most were seen in older individuals but were more likely to affect males. Eleven individuals exhibited hyperostosis, the most easily recognized indicator, usually located on the parietal bone; the hyperostotic region averaged 8 cm in diameter and 3.0 cm in height. Seven displayed lytic lesions with areas much smaller in size than the hyperostosis, and many had vascular changes. The other cases had indicators that varied greatly in terms of location and expression and included both sclerotic lesions and hollow areas. Several authors also suggested other possible causes of the lesions. The findings reflect the non-pathognomonic nature of the effects of meningiomas. However, given their likely frequency and potentially severe effects in ancient people, it is argued that they should be taken into consideration more frequently when performing differential diagnoses.

## 1. Introduction

Many contributors to the paleopathology and history of medicine argue that cancer is a disease of civilization and that tumors—malignant and benign—are only a recent burden on human health. These claims are problematic for many reasons [1], but meningiomas are an interesting exception to this supposed rule. Diagnosis of the condition in living patients ultimately depends on histology. This is seldom possible for paleopathologists, even when mummified soft tissue is recovered, and histology of hyperostotic bone is seldom a topic of interest to clinical pathologists. Although no modern mortality rates associated with untreated meningiomas could be identified because they are so amenable to surgery, Aufderheide and Rodríguez-Martín [2] (p. 251) have suggested that many such tumors would have resulted in death in the past, even if, like most modern meningiomas, they are not malignant. Despite being soft tissue tumors, meningiomas are observable on the human skeleton when they cause hyperostosis of adjacent cranial bone. This association accounts for their early characterization as a discrete kind of brain tumor [3]. Campillo [4] has also created an eight-point scale of bone lesions associated with meningomas that includes increased vascularization, lytic destruction, and thickening of cranial tables. 

Assuming they were the most common tumor of the central nervous system (CNS) in the past as they are today [5], meningiomas have been less commonly identified by paleopathologists than one might expect, despite the fact that an appreciable frequency of cases does leave skeletal lesions. Modern developments such as radiation and hormone replacement therapy may have increased their prevalence somewhat [6] (p. 376), [7] (p. 2) or changed their distribution by sex. Here, we discuss the identification of meningiomas in paleopathology and explore implications for their prevalence and ancient treatment. 

## 2. Historical Background 

### 2.1. Meningiomas in Early Paleopathological Studies

The first observations concerning meningiomas date to the early twentieth century. Neurosurgeon Harvey Cushing’s 1922 characterization of meningioma [8] and archaeologist George Grant MacCurdy’s [9] near-simultaneous discovery of a case from Paucarcaucha in ancient Peru are incorrectly linked in some sources on the history of paleopathology. This iconic case was identified by MacCurdy as an osteosarcoma. He does not discuss his criteria, and while he cites a Cushing elsewhere in his article, the citation is to the work of Frank Hamilton Cushing, an ethnographer. The paleontologist Roy L. Moodie [10] promptly called MacCurdy’s case and three others from Peru meningioma, citing Cushing’s work, but Moodie did not engage in a differential diagnosis of the associated hyperostosis. The Paucarcaucha plates are widely reproduced, and it is surely the iconic case of ancient cancer. However, it was not diagnosed properly as a meningioma until physicians Kenneth H. Abbott and Cyril B. Courville described it, as well as Moodie’s case from Chavina [10] in detail in 1939 [11]. Courville had trained with Dr. Harvey Cushing. More recent reviewers [12,13] have accepted their opinion despite the starburst or honeycomb pattern in the hyperostosis, and perhaps the question of osteosarcoma or malignant meningioma should be revisited. 

A decade later, the work of Dr. Mahomed Kamal Hussein describing remains from ancient Egypt [14] represents one of the most successful early collaborations between archaeologists and physicians, demonstrating how the integration of findings from both fields can contribute to a better understanding of diseases in the past and in the present. Hussein does not give a differential diagnosis for the two crania in which he identifies meningiomas, but he does explore the implications for the antiquity of cancers. Hussein’s cases are the same ones illustrated by Rogers [15]. Rogers discusses “honeycomb” organization of the hyperostosis in the Meydum case, and epilepsy and hemiplegia as likely in the Helwan case.

Given their symptoms—headaches, vomiting, seizures, visual and hearing impairment, muscular weakness, and personality changes [7] (p. 2)—meningiomas were devastating to their victims before Cushing pioneered a surgical remedy. In an engaging historical review of meningiomas, Cucu and colleagues [3] note that among the ancient Egyptians, at least two individuals with regions of cranial hyperostosis exceeding 4.5 cm have been recovered (citing Kamel Hussein’s cases [14], also discussed by Rogers [15]; they then note that although written records do not specifically mention this phenomenon, ancient treatment for similar conditions “included excision with a knife, local paste applications, burning with red-hot irons, spells, or leaving the swelling untreated” [3] (p. 38). It has even been argued that trepanation may have been one of the treatments for chronic headaches that often accompanied meningioma [16,17]. However, it was not until the early 17th century when the first description of a tumor appeared that had all the classic characteristics of a meningioma in the living patient as is seen in the medical literature [3].

None of these recent discoveries in paleogenomics has had an impact on the paleopathology of meningiomas as yet, but advances are happening so rapidly that optimism seems warranted. Perhaps the famous Elephant Man case should be revisited with this association in mind [18]. Conspicuous in their absence, viral infections are not implicated in the etiology of meningioma, and hence we cannot deploy the interesting argument that such cancers ought to have been more common in the past than they are in the present [1]. 

### 2.2. Diagnoses of Meningiomas in the Past 

How often should paleopathologists find meningiomas if we limit our expectations to those that cause hyperostosis? Recent estimates for hyperostosis occurring with adult meningiomas include 4.5% [19] and 7.4% [20], but much earlier studies report rates as high as 25% [8,21], perhaps because radiography played a more important role in discovering meningiomas in the first part of the twentieth century. Among juveniles, the rate is even higher, reaching 25–49% [22]. The rate of hyperostosis also varies with the type of tumor involved with up to half of *en plaque* tumors being associated with bone growth [19]. Location as well appears to be important since hyperostosis is seen in 60% of interosseous tumors [23]. Factors that promote hyperostosis involve vascular disturbance, irritation of cranial bone by the tumor, and trauma, but the most accepted explanation is an invasion of the bone by the tumor [22,24]. Genetic factors in hyperostosis include Proteus Syndrome, a rare condition characterized by overgrowth in several tissue systems [25,26], osteoprotegerin (OPG), bone morphogenetic protein (BMP), and insulin-like growth factor 1 (IGF-1) [20]. The neuropeptide somatostatin also plays a role [27]. It would be very interesting to see these factors explored in supposed cases of ancient meningioma.

After the early 20th century, paleopathology becomes more careful about differential diagnosis, and that is true of meningioma diagnoses. Despite the fact that meningiomas were likely a frequent CNS tumor in past populations and that a portion of these meningiomas left skeletal evidence, reports of the condition in ancient populations are not common. Campillo [4] notes only five cases in a review of 3000 crania from the Neolithic to Medieval periods in Spain. In his review of meningiomas, Anderson [28] identifies eight likely cases, Brothwell and Brothwell [29] discuss nine, and Cucu et al. [3] as well as Okonwo and Lewis [30] list eleven. The most recent contribution to that literature [13] depends on its description and review of meningiomas on Anderson’s excellent but dated review [28] and adds only one case for a total of twelve. The didactic literature in paleopathology is thus conservative regarding tumors, as we have reviewed elsewhere [31]. 

The credible identification of meningiomas in skeletal remain is challenging. In the paleopathological review literature, meningiomas are often not extensively addressed, possibly because they are not primary bone tumors, perhaps because they are often benign, but also possibly because there are no pathognomonic manifestations nor even a well-accepted suite of traits associated with their diagnosis in dry bone. Lytic lesions appear to be more common when the tumor is first developing [32] (p. 383), and destructive lesions on the inner table that do not exhibit spiculation also are more characteristic of meningiomas than other conditions [32] (p. 379). Campillo [33] compiled a series of bony changes, most vascular in nature, commonly associated with meningiomas that he argued could potentially be used in the diagnosis of the condition; he acknowledged that all these changes occur with other conditions as well. Meningiomas occurring at the cranial base are more likely to be osteosclerotic [32] (p. 378). Blastic responses are even more likely to occur as a bony response to the tumor [27] (p. 591), especially in the vault region. Steinbock [24] notes that the slow growth of the hyperostosis tends to result in well-ossified, regularly ordered bone; although both tables may be involved, the outer table is more often affected. However, our literature does not really accommodate the huge variability in documented meningiomas, e.g., [19].

A number of other conditions, including various genetic anemias such as thalassemia, leukemia, hemangiomas, Paget’s disease, and osteosarcoma, can also result in cranial hypertrophy. However, most of these conditions have identifying traits, such as the appearance of spicules of hypertrophic bone or concordant postcranial lesions, that allow a differential diagnosis to separate them from meningiomas [31]. Unfortunately, the descriptions of meningiomas in ancient populations are not always sufficiently detailed to permit such determinations and some of the purportedly affected individuals are no longer available for further evaluation. However, at least two cases that have been re-evaluated are no longer considered to be meningiomas [10,34].

## 3. Data Collection and Analysis

In order to explore meningiomas in the past, an online search for cases in the paleopathological literature was conducted. To be counted, it had to involve skeletal or mummified remains, be evaluated by an individual with paleopathological training, and have meningioma as the primary diagnosis or a small group of final diagnoses made. We found at least 43 cases of possible meningioma as well as two other cases that have been subsequently discounted as meningioma (Table 1). As may be seen, the dates of the cases span from the *Homo heidelbergensis* era [35] to a 19th-century poorhouse in New York [36]. When the demographics of the individuals are considered, the sex distribution is also unusual, being nearly the inverse of expectations [31]. Some 61% of the sample is comprised of males, whereas in studies of modern patients with meningiomas, females are twice as likely to be affected [37]. This discrepancy may reflect a well-documented systematic male bias among physical anthropologists and paleopathologists in assessing sex [38] or a vaguer systematic cultural bias against the burial of women from ancient mortuary sites [39], but the magnitude of the difference in meningioma diagnoses is still quite surprising. Perhaps cranial trauma was a relatively more important contributing factor in ancient meningiomas than it is today. Or perhaps we are misdiagnosing trauma with hematoma as meningioma, as Moodie [10] did in his Ancon case.

In contrast, the age distribution accords well with expectations based on modern samples [37] in that all of the aged adults but two were middle-aged older [31]. The identification of only one subadult with a meningioma also agrees with studies showing that meningiomas are a rare occurrence among children today [55]. However, we might anticipate actually finding more juvenile cases in earlier populations relative to modern frequencies for several reasons. Children are a much larger component of ancient death assemblages than of modern ones, albeit as a result of far higher mortality due to infectious diseases of childhood. They are also apparently more likely to have hyperostotic reactions [22], and the tumors may result in more extensive areas of bone being affected since they are more likely to be larger and of the en plaque type compared to those seen in adults [55,56,57]. Additionally, Brothwell and Brothwell [29] note that fewer adults in ancient times reached the older ages at which meningioma becomes more common. It is arguable that differential preservation of the more fragile bones of females and subadults may also be a major factor in the unexpected demographic distribution of the sample under consideration.

Another factor in the scarcity of reports of meningioma in children may be the extent to which paleopathologists have focused on malnutrition and infectious disease in childhood. Paleopathologists often discuss porotic hyperostosis as if it were a diagnosis rather than a description. Danforth and colleagues [31] point out two other case reports in addition to their own in which meningioma should have been considered in juveniles with exuberant porotic hyperostosis see [58,59], but neither of these skeletons is available for restudy. 

Some 33 of 43 case studies gave at least some description of the lesions. These cases reveal considerable variability in the features that paleopathologists consider important in the diagnosis of meningioma. One of the most commonly mentioned is a thickening of the cranial vault, reported in 13 cases; the bone affected was most frequently the parietal, although the frontal was also occasionally involved. As may be seen in Table 2, the area of the hyperostotic region varies greatly, ranging from 2.1 cm [47] to 16 cm [42] in maximum diameter with a mean of 8.2 cm (*n* = 8). The thickness varies extensively as well, ranging from 1.6 cm [48] to 4.5 cm [9,28,50,54] with a mean of 3.0 cm (N = 5). A few cases also mentioned certain osteoblastic processes being involved, including enostoses [33]. 

Lytic lesions were the next most common expression of the purported meningiomas, being seen in seven cases, all occurring on the vault except for one individual in whom it was in the basioccipital area [45] (Table 2). They were much smaller in size than the hyperostotic lesions, ranging from 0.6 to 2.4 cm in diameter [47]. Another case described a hollow lesion on the greater wing of the sphenoid [46]. The last group of changes involved various changes to the inner vault tables, including depressions of the parietal [35] and parasagittal regions [40], as well as large arachnoid granulations [46], irregular bone formation [41], and meningeal hypervascularization [16,33,47]. Several cases provided no description of the lesions involved.

Contrary to clinical data, the most commonly reported presentation of meningiomas in past populations involves hyperostosis, reflecting the lack of soft tissue for histology in individuals from whom only skeletal remains are preserved. Taking this bias into account, how frequent were meningiomas in the past? Many natural mummies from dry environments, for example, Peru, have been carefully autopsied with examination of soft tissue histology where possible. Gerszten et al. [60] found only ten tumors of any kind in their review of thousands of Andean mummies, suggesting that the recent origins of carcinogens account for this low incidence. Likewise, it is striking that only two meningiomas have been reported from ancient Egypt (Table 1). While some artificial mummification processes, for example, brain removal in ancient Egypt, might destroy soft tissue evidence, thus far we are aware of no cases of meningioma identified in mummified soft tissue. We discovered no diagnoses of meningioma from Egypt more recently than Rogers’s two cases [15]. This deficit is even more striking given the late Dr. Eugen Strouhal’s long interest in this region, as well as his documentation of 53 malignant tumors in mummies and skeletons [61]. Strouhal and Němečková [61] (p. 293) point out that “this geographical distribution is not a result of the real frequency of tumours in these various countries or number of their populations. They reflect the intensity of archaeological examination of cemeteries in single countries, the amount of uncovered and stored human remains, submitted to examination, and activity of the anthropologists, possessing knowledge how to identify tumours and study them, or hand them to palaeopathologists.” With all this attention, why are there still just two meningiomas from ancient Egypt? Perhaps Strouhal was less interested in benign tumors, whatever their consequences. However, a number of the ancient cases do present osteolytic changes, in particular increased vascularization [33]. Evaluating increased vascularization from skeletal evidence alone can be subtle, but potentially might result in overdiagnosis because of subjective criteria and confusion with normal features such as arachnoid granulations. It would be very useful to paleopathologists if someone were to review a large series of clinical cases with an eye to diameter, thickness, and location of any enostoses. Therefore, detailed description is especially important, although unfortunately often lacking (Table 2). 

For some of the meningioma cases identified in the paleopathological literature, other possible etiologies were offered by the publication authors. For example, Danforth and colleagues [31] consider other diagnoses unlikely rather than excluded. Pechenkina and colleagues [62] find angioma more likely than meningioma in their case, and angioma as well as unidentified tumor are considered as alternatives in a medieval case from Poland [63,64]. Ortner and Putschar [54] suggest that the lesion observed in their case study might also be an eosinophilic granuloma, carcinoma, or angioma. Alternate diagnoses such as these with careful description and weighing of relative likelihood is better science than the over-confidence adopted by an earlier generation of paleopathologists. Our literature would also benefit from more critique. Few diagnoses of meningioma have been re-evaluated and withdrawn. In addition to Moodie’s case from Ancon, Peru, reassessed by later scholars as post-mortem damage, a putative juvenile case in a fossil human from Lazaret in France has been recently withdrawn after careful restudy [34].

**Figure 1:**
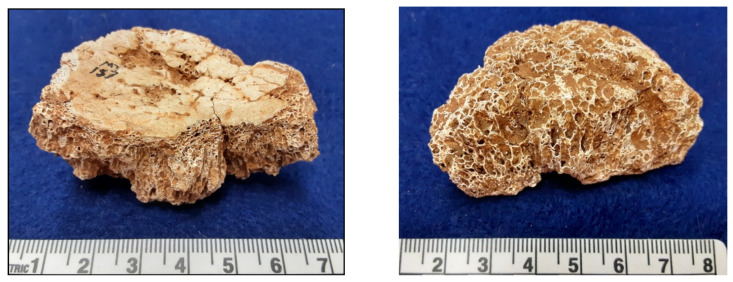
Parietal fragment from hyperostotic meningioma in 11–13-year-old Maya child from Tipu, Belize. (AD 1540–1638) [31]. (**Left**) View of thick diplöe and thin internal table. (**Right**) View of diplöe and poorly defined external table; red-brown material is soil in diplöic space.

We find ourselves making critical comments that apply to our own work as well, and we want to make amends here. We were disappointed that the morphology of the lesions in our juvenile meningioma case from Belize [31] was not clearer from the illustrations. Figure 1 shows another view of one of the fragments in which the thin inner table, columnar/hair-on-end structure of the hyperostosis, and minimal outer table are clearer. The case from the Koster site in Illinois—published by Cook [46] at a time when it was difficult to find a venue for case studies of rarities—is minimally described. This case is particularly interesting because a CAT study (performed by Dr. Mark Wisen) was critical in distinguishing meningioma from angioma/hemangioma. The lesion, a void in the greater wing of the sphenoid, was centered on the temporal fossa, not the margins of the basisphenoid, where many angiomas originate.

## 4. Conclusions

Identification of disease in the past is difficult even when there are written records and images, as Cucu and colleagues [3] demonstrate. The history of medicine regarding meningiomas is fraught with difficulties before Cushing’s landmark publication in 1922 [8] that provided the first definitive description of the condition. In the more distant past, the only evidence is usually found on the skeleton. As is frequently noted, the number of diseases that leave bony lesions is limited, and most of the lesions seen in bone are not clearly diagnostic. Paleopathologists may not always consider the entire range of possible etiologies: for example, the hyperostosis commonly associated with juvenile meningiomas may resemble porotic hyperostosis and may be mistakenly attributed to anemia [30]. In adults, careful differential diagnosis regarding trauma is important. We particularly single out the case studies of Pechenkina et al. [62] and Kornafel et al. [63] as models for what all of us in paleopathology should be doing.

Advances in paleogenomics and imaging have revolutionized the paleopathology of infectious diseases, and these techniques will undoubtedly help in diagnosing ancient meningiomas, but until these techniques become much more accessible, the collaboration of paleopathologists and clinicians will continue to be the most productive strategy to pursue. We hope that we can interest some of the readers of *Cancers* in collaborating.

## Figures and Tables

**Table 1:** Ancient Meningiomas in the Paleopathological Literature ^1^.

Location of Case	Sex	Age	Date	Ref.
Steinheim, Germany	F?	25–30 y	365,000 BP	[35]
Stetten ob Lontal, Germany	M	30–40 y	32,500 BP	[40]
Castellar, France	M	~50 y	Neolithic	[34]
Sant Quirze de Galliners, Spain	M	50 y	Neolithic	[16,33]
Cova d’Aigües Vives, Spain	F	Older adult	Neolithic	[16,33]
Barranc de Rifà Tarragones, Spain	?	Adult?	Neolithic	[33]
Roevejøj, Denmark	M	Adult	Neolithic	[41]
Linz, Austria	F	16–20 y	Early Bronze Age	[42]
Helouan, Egypt	M	40–60 y	ca. 3400 BC	[15]
Meydum, Egypt	M	50–80 y	1100–1200 BC	[15]
Hualcuy, Peru	M	40–50 y	2000 BP	[43]
Radley, England	F	Adult	Roman	[44]
Chaviña, Peru	F	Middle adult	Pre-European contact	[10]
Chicama, Peru	M	Adult	Pre-European contact	[10]
San Nicolas Island, CA, USA	M	Middle/older adult	Pre-European contact	[11]
Chernovski, AK, USA	M	~40 y	AD 1000–1800	[45]
Koster Md, Greene Co., IL, USA	F	Old adult	AD 1150–1350	[46]
Tarbat, Scotland, UK	M	Adult	Medieval	[29]
Reial Basilica de Sta Maria Mar, Spain	?	Adult?	Middle Ages	[33]
La Olmeda, Spain	?	Adult?	Middle Ages	[33]
Cherry Hinton, England, UK (10 ind)	?	Unreported	AD 800–1100	[47]
Czarna Wielka, Biylystok, Poland	M	Mature adult	AD 1100–1300	[48]
Stuttgart, Germany	M	Adult	AD 1100–1300	[49]
Sedlčany, Czech Republic	M	50+ y	AD 1298–1550	[17]
Rochester, England	F	35–50 y	AD 1300–1400	[28,50]
Vadstena, Sweden	F	~70 y	AD 1373	[51]
Tipu, Belize	?	11–13 y	AD 1560–1640	[31]
Iglesia Sta. Cruz y Soledad, de Nuestra Señora, Mexico	?	Adult?	Colonial	[52]
Erie County, NY, USA	F	Older adult	AD 1851–1913	[36]
Paucarchancha, Peru	M	Older adult	Not reported	[9,53]
St. Lawrence Island, AK, USA	M	45–65 y	Not reported	[54]
Hertfordshire, England, UK	M	Adult	Not reported	[47]
Hertfordshire, England, UK	F	45+ y	Not reported	[47]
Hertfordshire, England, UK	M	Adult	Not reported	[47]
Discounted cases:				
Le Lazaret, Nice, France	?	~9 y	200,000 BC	[34]
Ancon, Peru	F	Adult	Pre-European contact	[10]

^1^ Adapted from [31] (Suppl. 1).

**Table 2:** Lesion Characteristics of ancient meningiomas reported in the paleopathological literature ^1^.

Location of Case	Bone(s) Affected	Description of Lesion	Ref.
Cases with Hyperostosis:			
Tarbot, Scotland, UK	Frontoparietal	Hyperostosis is 3 cm in di; outer table is remodeled with bone destruction of inner table	[29]
Paucarchancha, Peru	Parietal	Hyperostosis is 14 cm × 11 cm, 4.5 cm in ht	[9,53]
Chaviña, Peru	Frontal, parietals	Hyperostosis is 2.0 cm in ht with slight hyperostosis on inner table	[10]
Chicama, Peru	Parietal	Hyperostosis is 10 cm in di with inner table also affected	[10]
Hualcuy, Peru	Temporal	Hyperostosis is 2.3 cm in di; inner table affected, enlarged mastoid cells and vascular spaces communicate with lesion	[43]
San Nicolas Is, CA, USA	Frontal, parietals	Erosion of inner table	[11]
Helouan, Egypt	Parietal	Both tables affected	[15]
Meydum, Egypt	Parietal	Lesion radiates from single site	[15]
Rochester, England, UK	Frontal, sphenoid	Hyperostosis is 6.5 cm × 6.3 cm, 4.5 cm in ht; osteolytic region on inner table and zygomatic	[28,50]
Tipu, Belize	Parietals	Hyperostosis is at least 180 sq cm, 2.5 cm in ht; inner table exhibits thinning and increased vascularization	[31]
Erie Co., NY, USA	Frontal	Hyperostosis is 3.7 × 4.4 cm; sclerotic portion containing active osteolytic area; osteoblastic growths on inner table	[36]
Stuttgart, Germany	Parietal	Hyperostosis is 9.2 × 7.5 cm, 1.6 cm in ht	[49]
Linz, Austria	Frontal	Hyperostosis is 14 × 16 cm; spiculated bone on outer table; inner table grooved, hyperostotic	[42]
Cases with Lytic Lesions:			
Hertfordshire, England, UK	Frontal, parietal	Lytic lesion is 2.4 × 1.1 cm; associated with arachnoid depressions and enlarged meningeal artery impression	[47]
Hertfordshire, England, UK	Parietal	Perforating lytic lesion on inner table is 2.1 × 2.1 cm; second lytic lesion on frontal is 1.4 × 0.7 cm; both associated with enlarged meningeal artery impressions	[47]
Hertfordshire, England, UK	Parietal	Lytic lesion on inner table 0.95 × 0.6 cm; enlarged meningeal artery impressions	[47]
St. Lawrence Island, AK, USA	Vault	Pumice-like texture on vault, face; two large osteolytic lesions on parietal	[54]
Chernovski, AK, USA	Basi-occipital	Osteolytic lesions of inner table extending into left maxilla, palatine	[45]
Sant Quirze de Galliners, Spain	Not reported	Irregular lytic lesion with perforation of outer table, enlarged meningeal vessels on inner table	[16,33]
Cases with Enostomas:			
Cova d’Aigües Vives, Spain	Frontal	Enostoma within squama, smaller area of hyperostosis on inner table	[16,33]
La Olmeda, Spain	Not reported	Enostoma with exocranial bulging	[33]
Reial Basilica de Sta. Maria del Mar, Spain	Not reported	Enostoma associated with enlarged meningeal artery	[33]
Vadstena, Sweden	Vertex	Elevation with little or no hyperostosis, deep endocranial indentation with vascularity	[51]
Sedlčany, Czech Republic	Frontal	Large arachnoid granulation along with wider and more branched meningeal vessels; condensation surrounding lesion	[17]
Barranc de Rifà Tarragonés, Spain	Not reported	Bilateral meningeal hypervascularization of inner table; possible meningioma of falx	[33]
Cases with Other Manifestations:			
Koster Md, Greene Co., IL, USA	Sphenoid	Hollow lesion on greater wing; shell of porous bone 5.7 × 4.1 cm on outer table	[46]
Roevejøj, Denmark	Occipital, parietals	Irregular bone formation with several perforations	[41]
Iglesia Sta. Cruz y Soledad de Nuestra Señora, Spain	Frontal	Osteogenic lesions in orbit and occipital squama	[52]
Stetten ob Lontal, Germany	Parasagittal	Depression; no hyperostosis	[40]
Steinheim, Germany	Parietal	Lesion 5.1 × 4.3 cm in di, 2.5 cm deep on inner table; surface is area is smooth and regular	[35]
Castellar, France	Vault	Meningioma among many other diagnoses considered	[34]
Radley, England, UK	Parietal	Slight endocranial changes; may be angioma	[44]
Cases with No Description of Lesion:			
Czarna Wielka, Biylystok, Poland	Not reported	No description provided	[38]
Cherry Hinton, England, UK	Not reported	10/683 individuals in population with lesions; no descriptions provided	[47]
Discounted Cases:			
Ancon, Peru	Not reported	“Early…hyperostosis due to a meningioma”; now attributed to postmortem erosion	[10]
Le Lazaret, Nice, France	Parietal	Hyperostosis 9.0 × 7.0 cm, endocranial vascularity; recently re-evaluated to be post-traumatic	[34]

^1^ Adapted from [31] (Suppl. 1).
